# Antitumor Activity of 2,9-Di-*Sec*-Butyl-1,10-Phenanthroline

**DOI:** 10.1371/journal.pone.0168450

**Published:** 2016-12-29

**Authors:** Dongsheng Wang, Shifang Peng, A. R. M. Ruhul Amin, Mohammad Aminur Rahman, Sreenivas Nannapaneni, Yuan Liu, Dong M. Shin, Nabil F. Saba, Jack F. Eichler, Zhuo G. Chen

**Affiliations:** 1 Department of Hematology and Medicinal Oncology, Winship Cancer Institute, Emory University School of Medicine, Atlanta, GA, United States of America; 2 Department of Biostatistics and Bioinformatics, Biostatistics and Bioinformatics Shared Resource at WCI, NE, Atlanta, GA, United States of America; 3 Department of Chemistry, University of California-Riverside, Riverside, CA, United States of America; University of South Alabama Mitchell Cancer Institute, UNITED STATES

## Abstract

The anti-tumor effect of a chelating phen-based ligand 2,9-di-*sec*-butyl-1,10-phenanthroline (dsBPT) and its combination with cisplatin were examined in both lung and head and neck cancer cell lines and xenograft animal models in this study. The effects of this agent on cell cycle and apoptosis were investigated. Protein markers relevant to these mechanisms were also assessed. We found that the inhibitory effect of dsBPT on lung and head and neck cancer cell growth (IC_50_ ranged between 0.1–0.2 μM) was 10 times greater than that on normal epithelial cells. dsBPT alone induced autophagy, G1 cell cycle arrest, and apoptosis. Our *in vivo* studies indicated that dsBPT inhibited tumor growth in a dose-dependent manner in a head and neck cancer xenograft mouse model. The combination of dsBPT with cisplatin synergistically inhibited cancer cell growth with a combination index of 0.3. Moreover, the combination significantly reduced tumor volume as compared with the untreated control (p = 0.0017) in a head and neck cancer xenograft model. No organ related toxicities were observed in treated animals. Our data suggest that dsBPT is a novel and potent antitumor drug that warrants further preclinical and clinical development either as a single agent or in combination with known chemotherapy drugs such as cisplatin.

## Introduction

Cisplatin has been widely used over the past 30 years to successfully treat a variety of cancers, including testicular, ovarian, head and neck, and bladder cancers [1–4]. Despite having shown clinical success, cisplatin does have drawbacks, particularly the fact that patients routinely experience severe side effects during treatment, and tumors often develop resistance to the drug [5–7]. In a broader ongoing effort to develop gold-based therapeutic alternatives for cisplatin, chelating 2,9-dialkyl-1,10-phenanthroline (^R^phen) ligands have been used to synthesize gold (III) coordination complexes possessing enhanced biological stability. Several recent studies have addressed the antiproliferative potential of 1,10-phenanthroline (phen)-based ligands and their metal complexes in other types of cancer cells [8–11]. Previous studies have shown phen-based ligands also exhibit anti-proliferation properties when not coordinated to the gold metal center *in vitro* [12–15]. Therefore, the current study aimed to determine the anti-proliferative activity of 2,9-di-*sec*-butyl-1,10-phenanthroline (dsBPT). Augmenting our recent report in which this compound was tested for activity against glioblastoma tumor [16], the work reported here describes testing of this compound against head and neck and lung tumor cell lines and reports the first data in regard to a combined therapy regime with this agent providing new insight into the potential antitumor mechanism of dsBPT.

In this study, the dsBPT chelator was evaluated in a series of *in vitro* and *in vivo* antitumor studies in a variety of head and neck and lung tumor cell lines. Cell viability assays revealed that dsBPT possessed significant anti-proliferation activity against head and neck and lung cancer cell lines, including one tumor cell line that has been previously shown to be resistant to cisplatin. These results prompted the investigation of the antitumor properties of dsBPT against mouse xenograft tumors generated from two of the cancer cell lines. Additional drug effect studies and cell signaling pathway analyses were carried out in order to gain insight about the possible mechanism through which this drug induces tumor cell death. Finally, a combination treatment comprised of dsBPT and cisplatin was evaluated in both *in vitro* tumor cell cultures and tumors grown in mouse models. The results of the assessment of the *in vivo* antitumor activity of dsBPT, the *in vivo* antitumor activity of the dsBPT-cisplatin combination therapy, and the potential tumor cell death pathways initiated by this compound are described herein.

## Materials and Methods

### Synthesis of dsBPT

dsBPT was synthesized and purified according to a previously published protocol [15]. The purity of the final product was verified by thin layer chromatography (TLC), 1H NMR, and elemental analysis. Elemental analyses were carried out by Atlantic Microlab, Inc (Norcross, GA). The theoretical ratio are C = 82.15% and H = 8.27%. Experimental ratio from the elemental analyses are C = 81.57% and H = 8.33%.

### Tumor cell lines

All head and neck squamous cancer cell lines (HNSCC) were maintained in DMEM/F12 (1:1) medium supplemented with 10% heat-inactivated fetal bovine serum in a 37°C, 5% CO_2_ humidified incubator. The HNSCC cell lines Tu212 and Tu686 were kindly provided by Dr. Gary L. Clayman (University of Texas MD Anderson Cancer Center, Houston, TX) in 2002 and Dr. Peter G. Sacks (New York University College of Dentistry, New York, NY) in 2013, respectively [17]. The human lung cancer cell lines A549 and H1703 used in this study were obtained from the laboratory of Dr. Shi-Yong Sun (Winship Cancer Institute of Emory University) [18]. These two lung cancer cell lines were maintained in RPMI 1640 media supplemented with 5% FBS. The immortalized bronchial epithelial cell line BEAS-2B was obtained from the laboratory of Dr. Xingming Deng (Winship Cancer Institute of Emory University) and maintained in DMEM with 10% FBS [19]. All cells were routinely screened for mycoplasma contamination by the MycoAlert Mycoplasma Detection Kit (Lonza Ltd., Allendale, NJ). The authenticity of cell lines Tu212, Tu686, and BEAS-2B was verified through genomic short tandem repeat (STR) profiling by the Research Animal Diagnostic Laboratory, University of Missouri (Columbia, MO) in September 2009, and by the Emory University Integrated Genomics Core (EIGC) in October 2013, respectively. The authenticity of A549 cells was examined by Dr. Sun [20]. H1703 cells were not checked for authenticity by the authors or the cell line provider.

### SRB cell growth assay and calculation of IC_50_

Sulforhodamine B (SRB) cytotoxicity assays were performed as described by Skehan *et*. *al*.[21]. Cells were seeded in 96-well plates at a density of 4000 cells/well overnight. Subsequently, the anti-cancer agent was added with concentrations ranging from 0–25 μM, and cells were incubated for another 72 hours. The cells were then fixed for 1 hour with 10% cold trichloroacetic acid, followed by washing with water. The cells were air-dried and stained with 0.4% SRB for 10 minutes followed by washing with 1% acetic acid and air-drying. The bound SRB was dissolved in 10 mM unbuffered Tris base (pH 10.5) and the plates were read with a microplate reader by detecting the absorbance of the sample at 492 nm. The percent survival was then calculated based upon the absorbance values relative to untreated samples. IC50 value was calculated with CalcuSyn (Biosoft, Ferguson MO)

### Flow cytometry

Cell apoptosis and cell cycle analyses were performed by flow cytometry. A549 and Tu212 cells were exposed to 8 μM and 4 μM dsBPT, respectively. Cells were harvested at 24, 48, and 72 h, and cell apoptosis was determined by Annexin-5 staining method using a Cell Lab Quanta^TM^ SC flow cytometer (Beckman Coulter). For cell cycle analysis, cells were exposed to equal concentrations of dsBPT and cisplatin (2 μM for A549 and 1 μM for Tu212) and stained with PI 24h and 48h after drug treatment.

### Western blot analysis

Whole cell lysates were extracted from drug-treated cells using lysis buffer containing 50mmol/L HEPES buffer, 150mmol/L NaCl, 1mmol/L EDTA (pH 8.0), 1mmol/L EGTA (pH8.0), 1% IGEPAL CA-630, 0.5% Triton X-100, 10mmol/L NaF, 2mmol/L Na_3_VO_4_, 10mmol/L β-glycerophosphate and 1% Protease Inhibitor Cocktail (Sigma-Aldrich, St Louis, MO). Protein (20–30 μg) was separated on 8–12% SDS-PAGE, transferred onto a polyvinylidene difluoride membrane (Millipore Corp., Billerica, MA) and the desired protein was probed with specific antibodies–DR5 (Prosci Incorporated, Poway, CA), p21 (Santa Cruz Biotechnology Inc, Santa Cruz, CA), p27 (Santa Cruz Biotechnology, Inc, Santa Cruz, CA), caspase 3 (BD Pharmingen, San Jose, CA), and caspase 8 (Santa Cruz Biotechnology Inc, Santa Cruz, CA), Mouse anti-actin antibody (Trevigen, Gaithersburg, MD) was used as a sample loading control. Immunostained protein bands were detected with an enhanced chemiluminescence kit (Thermo Scientific, Rockfield, IL).

### Ki67 immunohistochemistry (IHC) and TUNEL staining

To test the cytotoxicity of dsBPT in tumor cells, xenografted tumor tissues were fixed in paraformaldehyde and subjected to paraffin embedment. Embedded samples were cut to a thickness of 8μm. IHC staining of Ki67 was performed using the R.T.U. Vectastain kit following the manufacturer’s standard protocol (Vector Laboratories, Burlingame, CA). Tissue sections were incubated with mouse anti-human Ki-67 antibody (Biomeda, Foster, CA) overnight at 4°C. The slides were stained with 3,3 diaminobenzidine (Vector Laboratories, Burlingame, CA) and counterstained with hematoxylin (Vector Laboratories, Burlingame, CA). For TUNEL assay, frozen tumor samples were kept in OCT, and assayed using the TUNEL kit (Promega, Madison, WI) following the manufacturer’s procedure. The slides were counterstained with DAPI (Invitrogen, Eugene, OR).

### Nude mouse xenograft model

All animal experiments were approved by the Institutional Animal Care and Use Committee (IACUC) of Emory University. Nude mice (athymic *nu/nu*, Taconic, NY), aged 4–6 weeks (~20 g body weight), were used to test the *in vivo* effects of dsBPT. Tu212 or A549 cells (2 x 10^6^) were injected into the right flank of the mice subcutaneously. The tumor volume was calculated using the formula: *V* = π/6 x larger diameter x (smaller diameter)^2^, as reported previously [22]. Growth curves were plotted using average tumor size within each experimental group at the defined time points.

Mice injected with tumor cells may develop illness symptoms, such as cachexia, respiratory distress, hunching over, ataxia, inability to mobilize around cage, or inability to either eat or drink. Mice were examined twice per week to determine the Tumor Burden Score established by the Emory IACUC. In any of the following cases, mice were euthanized by CO_2_ gas or other methods: (1) weight > 25% (Score = 18); (2) weight loss > 20 (Score >15, but < 18) plus decreased activity, or hunched posture (Score = 3); (3) epithelial tumor > 2.0 cm in length (Score > 12) plus decreased activity, or hunched posture (Score = 3); (4) epithelial tumor > 1.8 (Score = 12) and weight loss = 15% (Score = 12). Mice were euthanized with CO_2_ in a chamber that allows for their visualization, using a flow rate of 10–30% of the chamber volume per minute as controlled with a pressure-reducing regulator and a flow meter. All animals were checked for lack of breathing for at least 5 minutes prior to being placed in carcass cooler.

Two sets of animal experiments were performed. First, animals were treated with different doses of dsBPT, including 2 mg/kg, 5 mg/kg, and 10 mg/kg and the PBS control. The animals were continuously administered the agents 3 times a week by tail vein injection. Tumor size and animal weight were measured 3 times every week. In the second experiment, the combination effect of dsBPT and cisplatin was examined. The experimental groups were (i) PBS control, (ii) 2 mg/kg cisplatin, (iii) 2 mg/kg dsBPT, and (iv) 2 mg/kg of both cisplatin and dsBPT. Drugs were given intravenously every 6 days. Tumor size and animal weight were measured every 3 days. When the tumor size of the control group reached the IACUC endpoint, the animals were sacrificed, and the tumors were harvested for Ki67 IHC and TUNEL staining. Major organs were also harvested for H&E staining to evaluate the potential toxicity of the drug. For drug toxicity assays, mouse blood was subject to biochemical tests for alanine aminotransferase (ALT), aspartate aminotransferase (AST), creatinine and blood urea nitrogen concentrations. Samples were sent to the Clinical Pathology Laboratory, College of Veterinary Medicine, University of Georgia for these tests.

### Statistical analysis

The mice were randomly assigned to different treatment groups (described above) and control groups. The mixed effects model was mainly used to examine the tumor growth difference between each of the treatment groups and control. The fixed-effect terms in the model were groups, time, and interaction between groups and time, which allows different tumor growth rate by groups. The within subject covariance structure was chosen based on AIC and BIC criteria (the smaller, the better). Box-Cox data transformation was applied to the outcome to ensure the fitted model met statistical assumptions. Statistical analysis was performed using SAS 9.2. The critical significance level of 0.05 was chosen.

## Results

### *In vitro* anti-cell growth activity of dsBPT

**Fig 1:**
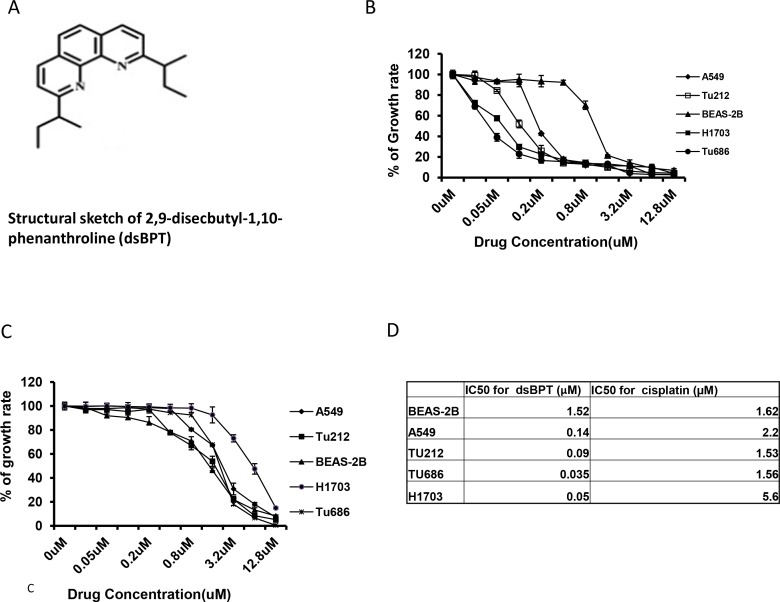
dsBPT inhibits cancer cell growth more potently than cisplatin *in vitro*. (A) Chemical structure of dsBPT. (B) SRB cytotoxicity assays were performed to detect survival of lung tumor cell lines H1703 and A549 and two HNSCC cell lines Tu212 and Tu686 treated with increasing doses of dsBPT. The immortalized bronchial epithelial cell line BEAS-2B was included as a control. (C) The commonly used chemotherapeutic drug cisplatin was also tested in parallel as a reference. (D) IC_50_ of dsBPT and cisplatin.

The structure of dsBPT is illustrated in Fig 1A. To determine the in vitro antitumor activity of dsBPT, SRB cytotoxicity assays were performed initially using two lung cancer cell lines, H1703 and A549 and two HNSCC cell lines, Tu212 and Tu686. The commonly used chemotherapeutic drug cisplatin was also tested in parallel as a reference. It was found that dsBPT had IC50 values ranging from 50–200 nM in these lung cancer and HNSCC cell lines (Fig 1B), representing a 20–100–fold higher efficacy than cisplatin (Fig 1C). dsBPT and cisplatin were also used to treat an immortalized non-tumorigenic bronchial epithelial cell line (BEAS-2B). dsBPT inhibited growth of this cell line at higher concentrations than the cancer cell lines with IC50 around 1.5 μM (Fig 1D). Conversely, cisplatin inhibited growth of both BEAS-2B and the cancer cells at similar concentration ranges. Finally, it was noted that the IC50 of dsBPT was over 100 times lower than that of cisplatin for the H1703 cancer cell line which is insensitive to cisplatin.

### dsBPT inhibits tumor cell growth by inducing cell cycle arrest, and apoptosis

**Fig 2:**
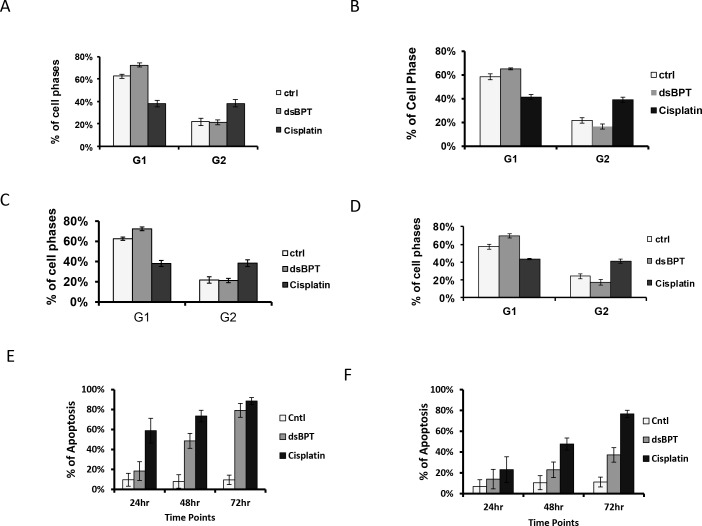
dsBPT alters cell cycle and induces apoptosis in cancer cells. Alteration of cell cycle was observed in A549 cells after 1 μM dsBPT treatment at 24 hours (A), 48 hours (C) and in Tu212 cells at 24 hours (B), 48 hours (D). dsBPT induced cell apoptosis at concentrations of 4 μM in a time-dependent manner in both A549 and Tu212 cell lines (E and F).

To identify the mechanism of cell inhibitory effect of dsBPT, we examined possible alterations in cell cycle distribution and protein markers relevant to cell cycle regulation and cell growth arrest. It was found that treatment with dsBPT at 1–2 μM concentrations resulted in significantly higher levels of G1 cell cycle arrest than seen following cisplatin treatment in both A549 and Tu212 cancer cell lines at both 24 and 48 hour time points (p< 0.05), while cisplatin significantly induced G2 cell cycle arrest (p <0.05) at 24 and 48 hour time points (Fig 2A–2D).

Flow cytometry was used to quantify the level of apoptosis in A549 and Tu212 cancer cells upon treatment with dsBPT. We did not observe cell apoptosis when the same concentrations (1–2 μM) as used in cell cycle analysis were applied (S1 Fig). However, we found that dsBPT at concentrations of 4–8 μM induced cell apoptosis in a time-dependent manner in both A549 and Tu212 cell lines (Fig 2E and 2F). dsBPT induced apoptosis in the A549 tumor cell line at levels comparable to cisplatin at 72 hours, but not at lower levels at 24 and 48 hours (Fig 2E). In the Tu212 tumor cell line, dsBPT induced lower levels of apoptosis compared to cisplatin at all 3 time points (Fig 2F).

### dsBPT alters cellular regulation pathways

**Fig 3:**
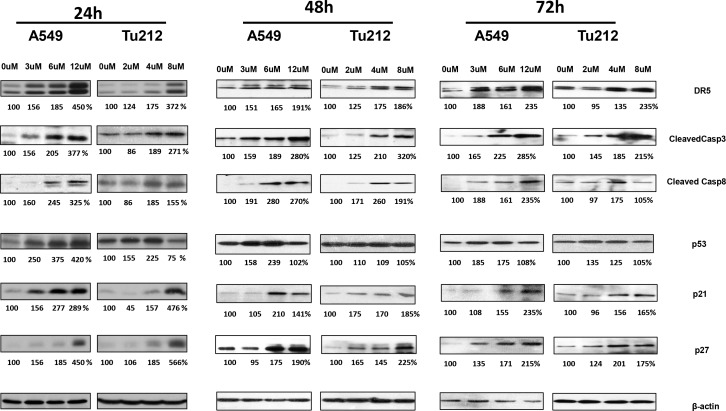
dsBPT alters cellular regulation pathways. Increased levels of caspase 3, and caspase 8 were seen in A549 and Tu212 tumor cell lines when treated with dsBPT for 24, 48 and 72 hours. DR5 expression levels were increased in both cancer cell lines upon treatment with dsBPT. p53 was induced in A549 cells, but not in Tu212 cells which have mutated p53. Expression levels of cyclin-dependent kinase inhibitors p21 and p27 were induced in both cancer cell lines by dsBPT compared to untreated control. Quantification is presented as percentage change of the expression level normalized to the control. Data represent 3 repeated experiments.

In order to identify the mechanism underlying the induction of apoptosis and cell cycle arrest by dsBPT, we carried out Western blot analyses to detect alterations in signaling pathways relevant to the regulation of cell cycle progression and apoptosis in A549 and Tu212 cell lines after treatment with the drug for 24, 48 and 72 hours. Increased levels of caspase 8 were found in both A549 and Tu212 tumor cell lines when treated with dsBPT (Fig 3). Given that caspase 8-mediated apoptosis is driven by the induction of DR5, we examined the levels of DR5 and found that the expression level of this protein also increased in both cancer cell lines upon treatment with dsBPT. Expression of p53 was clearly induced in A549 cells, but not in Tu212 cells, which carry mutated p53. As G1 cell cycle arrest was observed after treating A549 and Tu212 cells with dsBPT, the cyclin-dependent kinase inhibitors p21 and p27 were examined, and were found to have higher expression levels in both cancer cell lines than the untreated control (Fig 3). The trend of the drug effect on examined proteins is basically consistent with the cell cycle alteration and apoptosis from the earlier to the later time points. We also found that dsBPT treatment resulted in higher LC3 expression, a biomarker for induction of autophagy, in both the A549 and Tu212 cell lines (S2 Fig). These protein alterations confirmed that cell cycle arrest and apoptosis were induced by dsBPT in a dose dependent manner. The underlying mechanism of the anti-tumor effect of dsBPT involves several different cellular regulatory pathways.

### dsBPT inhibits Tu212 xenograft tumor growth in nude mice

**Fig 4:**
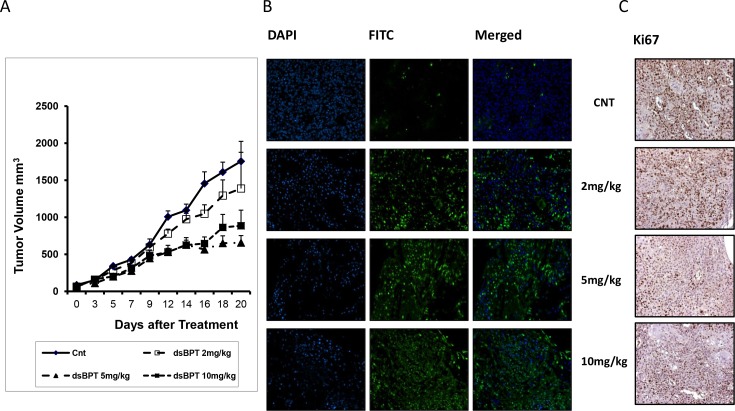
dsBPT inhibits Tu212 xenograft tumor growth in nude mice. (A) dsBPT significantly inhibited Tu212 xenograft tumor growth at doses of 5mg/kg and 10 mg/kg as compared with the control (both doses p<0.04). (B and C) IHC and TUNEL analyses of the xenograft tissues showed that dsBPT reduced levels of the proliferation marker Ki67 and induced apoptotic marker TUNEL signal.

dsBPT was initially used as single agent to treat mouse xenograft tumors originating from the Tu212 cell line. Mice were injected with 2×10^6^ Tu212 cells. dsBPT was administered by tail vein injection at 0mg/kg, 2mg/kg, 5mg/kg, and 10mg/kg doses. One group of mice treated with PBS was also included as a control. Each group included 6 mice. Tumor volume and mice body weight were measured every 3 days. dsBPT significantly inhibited Tu212 xenograft tumor growth at doses of 5mg/kg and 10 mg/kg as compared with the control (both doses p<0.04) (Fig 4A). IHC and TUNEL analyses of the xenograft tissues showed that dsBPT reduced the proliferating marker Ki67 and induced apoptotic marker TUNEL signal (Fig 4B and 4C). There was no non-specific toxicity to mice, indicated by the lack of abnormal liver/kidney functions (Table 1).

**Table 1:** Liver and Kidney Function of Mice Treated with dsBPT.

Group	Urea Nitrogen(mg/dL)	Creatinine (mg/dL)	ALT (U/L)	AST (U/L)
CNT	31	0.2	24	91
	18	0.2	19	66
	26	0.2	23	70
				
				
2mg/kg	27	0.2	35	162
	26	0.2	57	126
	15	0.2	23	156
				
				
5mg/kg	24	0.2	25	121
	25		24	192
	16	0.2	18	76
				
				
10mg/kg	23	0.2	19	66
	12	0.2	29	242
	26	0.2	33	144
				

### Combination of dsBPT and cisplatin has a synergetic antitumor effect *in vitro* and *in vivo*

**Fig 5:**
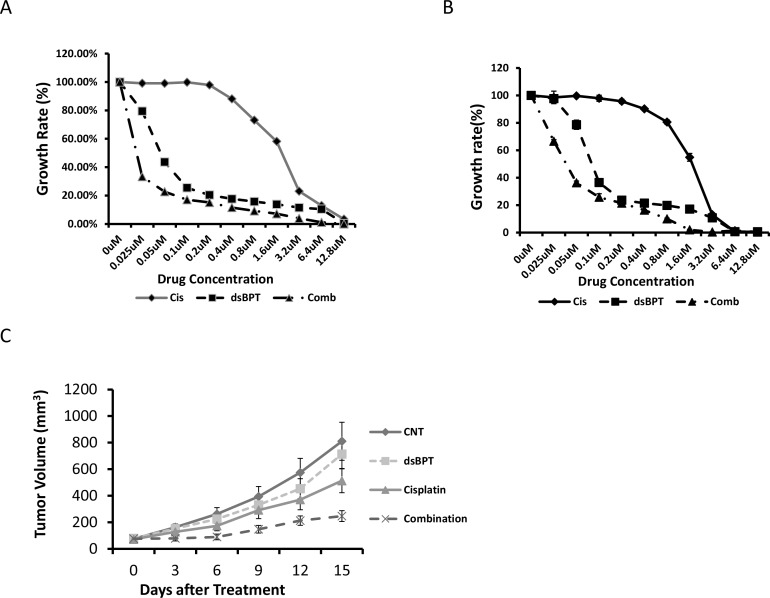
Combination of dsBPT and cisplatin has a synergetic antitumor effect *in vitro* and *in vivo*. (A and B) Combination of dsBPT with cisplatin synergistically enhanced the inhibitory activity of both drugs *in vitro* in A549 cells (A) and in Tu212 cells (B). (C) Combination of dsBPT with cisplatin also more potently inhibited tumor growth in the Tu212 xenograft mouse model.

**Fig 6:**
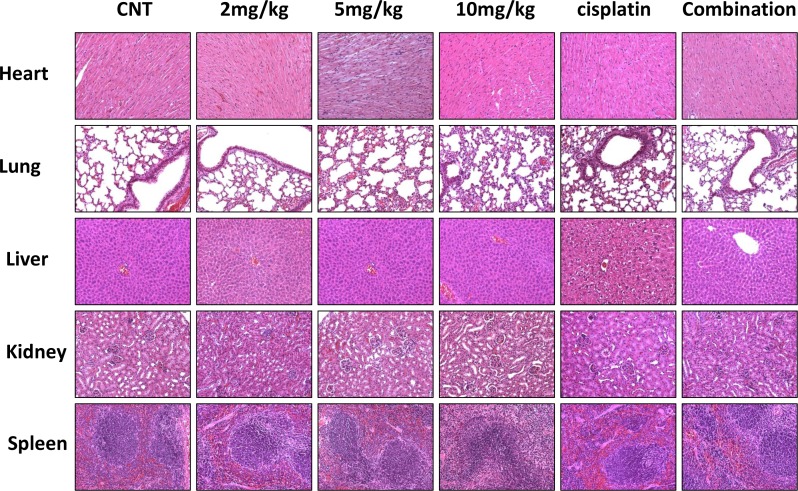
Toxicity study of dsBPT in mice. No organ toxicity was seen following treatment with different doses of dsBPT alone or in combination with 2mg/kg cisplatin in mice. Representative IHC images show 1 out of 6 mice.

Since dsBPT induces an overall lower rate of apoptosis than cisplatin, but induces cell cycle arrest at different stages from cisplatin at a relatively low concentration (1–2 μM), we speculated that combination treatment using both dsBPT and cisplatin may increase the tumor killing effect. As expected, the combination of dsBPT with cisplatin synergistically enhanced the inhibitory effect of both drugs as indicated by CIs of less than 1.0 (Fig 5A and 5B and S3 Fig). As expected, the combination induced higher levels of apoptosis than either of the single drugs at a concentration of 1–2 μM in both cancer cell lines (S1 Fig). This observation was confirmed by the second set of animal studies, in which 2 mg/kg dsBPT and 2 mg/kg cisplatin were given as single agents or in combination to different groups of mice (n = 6). The combined treatment significantly inhibited tumor growth as compared with the control (p = 0.0017) and the two single drugs (p <0.01) (Fig 5C). Examining the tissues of major organs, such as heart, lung, liver, kidney, and spleen, of the mice showed no damage to these organ tissues after both *in vivo* experiments (Fig 6). In addition, we did not observe any loss in bodyweight (S4 Fig), suggesting no general toxicity of dsBPT and its combination with cisplatin at the doses used.

## Discussion

We previously reported the antitumor effect of dsBPT on a glioblastoma cell line [16]. In the current study, we extended our investigation of this compound to HNSCC and lung cancer cells and provide the first data in regards to a combination therapy regime with this drug. Our results clearly show that dsBPT had significantly lower *in vitro* toxicity toward a normal cell line compared to the currently used drug cisplatin, and that its IC50 values in cancer cell lines were 20–100 times lower than that of cisplatin. These results prompted the investigation of this drug in an *in vivo* animal model, which confirmed the anti-tumor activity observed *in vitro*, indicating the potential of this agent as an anticancer therapy.

dsBPT was initially used as a chelater to form a gold complex [12]. In fact, dsBPT itself has a stronger inhibitory effect on cancer cell growth than its gold complex. Using chelaters as anticancer agents has been reported in the past years [23–25]. Cancer cells have greater requirements than normal cells for essential metal ions such as iron, copper, and zinc for growth and proliferation. Chelators, therefore, can inhibit cancer cell growth through the control of metabolic proteins involved in the regulation or utilization of these metals. For example, curcumin inhibits cancer cells partially through iron chelation [26]. In addition, proteins that control the concentration and function of metal chelators were reported to induce apoptosis in cancer cells. Greene et al., found that treatment with the iron chelators tachpyridine, dipyridyl, and desferrioxamine activated caspases 9, 3, and 8 [27].

In addition to chelating gold ion, we found that dsBPT can form a complex with iron, zinc, and copper ions (data not shown). Although the exact target of dsBPT has not been identified, this agent clearly induces apoptosis (Fig 2E and 2F), which is also demonstrated by the cleavage of caspase 8, and 3, (Fig 3). Our data showing dose-dependent upregulation of DR5 also suggest that the extrinsic death pathway might be involved in dsBPT-mediated apoptosis.

We observed that dsBPT did not induce apoptosis at lower concentrations (1–2 μM), rather, it blocked cell cycle progression (Fig 2A–2D) by activation of p21 and p27 in both lung and head and neck cancer cell lines (Fig 3). We did cell cycle analysis only in 24 and 48 hours because most cells died at 72 hours, which could not provide an accurate cell cycle distribution. Cell cycle arrest was also evidenced by the dose-dependent induction of p53 in A549 cells, but not in Tu212 cells which had mutated p53. It was interesting to find that dsBPT induced the autophagy marker LC3 in a dose-dependent manner in both lung and head and neck cancer cells. It is possible that autophagy was caused by metal chelation. Hancock *et al*, reported that a copper chelate of thiosemicarbazone NSC 689534 induced oxidative/ER stress and inhibited tumor growth *in vitro* and *in vivo* partially through autophagy [28]. Several research groups have also reported that iron chelation could result in autophagy and cell death [29–31]. The mechanism by which dsBPT induces autophagy deserves further investigation.

Since dsBPT can inhibit cancer growth but cannot cause cell death at a relatively low concentration, we tested whether combining this agent with a cytotoxic agent, such as cisplatin, can induce cancer cell death. Cisplatin is a highly effective antineoplastic drug, but it causes dose-dependent side effects that compromise its therapeutic efficacy. When the administration dose exceeds 50 mg/m^2^, cisplatin can induce severe nephrotoxicity [32]. Furthermore, 90% of patients who receive a cumulative dose of cisplatin exceeding 300 mg/m^2^ show severe signs of neuropathy [33]. One way to circumvent the side effects of cisplatin is to use it at a lower dose in a combination therapy. Our results clearly show that the combination of dsBPT and lower dose cisplatin have a synergetic therapeutic effect. The significant improvement in efficacy seen with the simultaneous treatment of these two drugs compared to the single drugs suggests a promising future for this new combined therapy.

## Supporting Information

S1 Fig Apoptosis assay for A549 and Tu212 cancer cells with both single and the combined treatment. No significant cell apoptosis was observed when the concentration 1–2 μM of dsBPT and cisplatin as used in cell cycle analysis were applied to both A549 (A), and Tu212 (B) cell lines compare to vehicle control. However, the combination of the two at the concentration of 1–2 μM induced higher apoptosis than any of the single drugs at the same concentration in both cancer cell lines (A and B). (Figure represents 3 different tests).
(PDF)

S2 Fig Western blot analysis of autophagy biomarker. dsBPT treatment induced LC3 expression in both A549 and Tu212 cell lines. (SE stands for short exposure, LE stands for long exposure).
(PDF)

S3 Fig Combination Index of the combined treatment with cisplatin and dsBPT. dsBPT with cisplatin synergistically enhanced the inhibitory sensibility of the both drugs as indicated by CIs which are less than 1.0. Figure is generated using CalcuSyn 2.0 software (Biosoft).
(PDF)

S4 Fig Body weight changes of the mice treated with dsBPT, cisplatin, and their combination. No bodyweight loss was observed when mice were treated with 2mg/Kg dsBPT, cisplatin, and combination of the two agents with the same concentration.
(PDF)
