# Supplementary material for: Antitumor Activity of 2,9-Di-Sec-Butyl-1,10-Phenanthroline
Source: PLoS One. 2016 Dec 29;11(12):e0168450. doi: 10.1371/journal.pone.0168450 (PMC5199049; doi:10.1371/journal.pone.0168450)
Supplement: S1 Fig — No significant cell apoptosis was observed when the concentration 1–2 μM of dsBPT and cisplatin as used in cell cycle analysis were applied to both A549 (A), and Tu212 (B) cell lines compare to vehicle control. However, the combination of the two at the concentration of 1–2 μM induced higher apoptosis than any of the single drugs at the same concentration in both cancer cell lines (A and B). (Figure represents 3 different tests). (PDF) [file pone.0168450.s001.pdf]

A

A549 (Cisplatin 2uM, dsBPT 2uM, Combination)

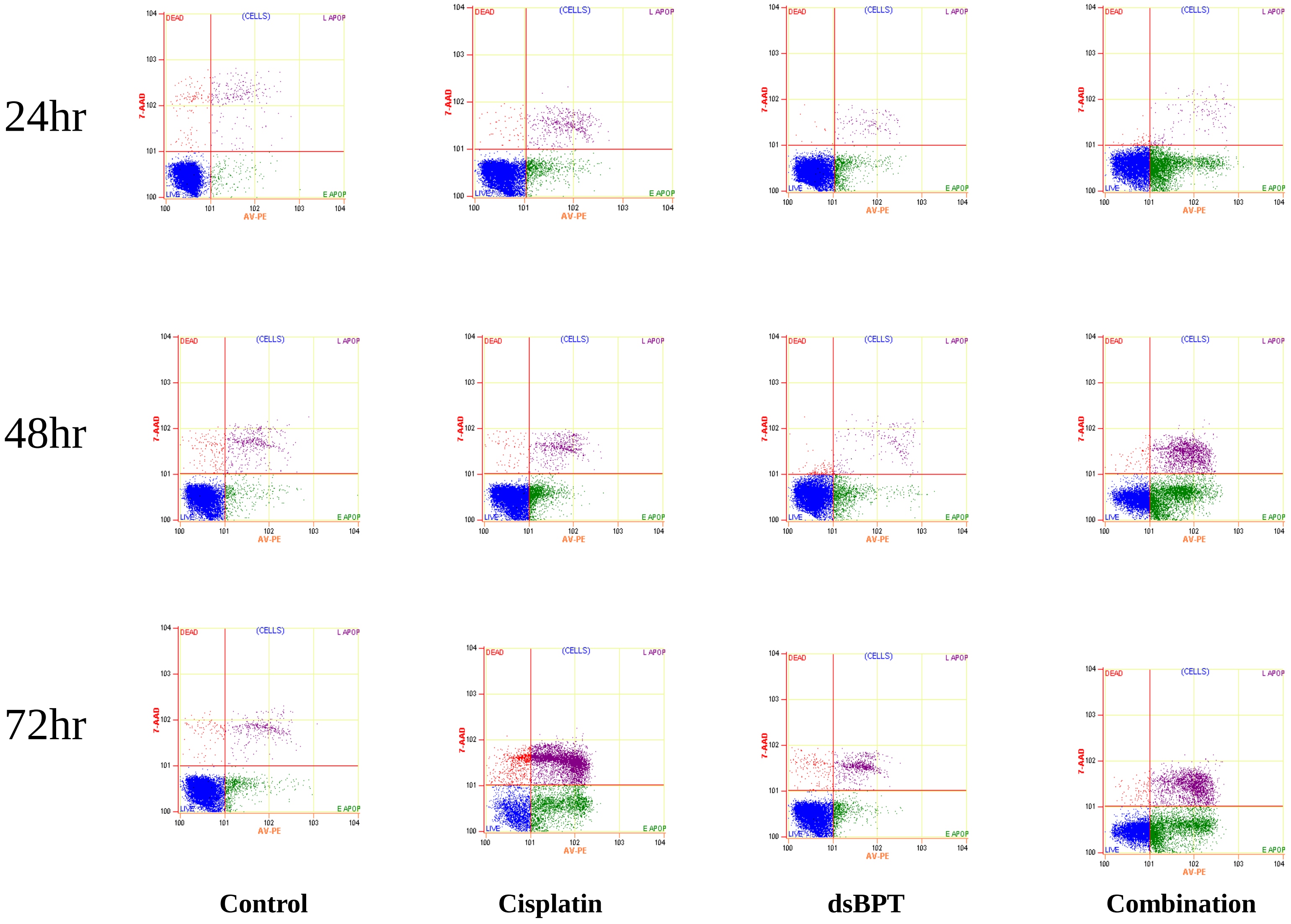

Supplementary Fig S1

B

Tu212 (Cisplatin 1uM, dsBPT 1uM, Combination)

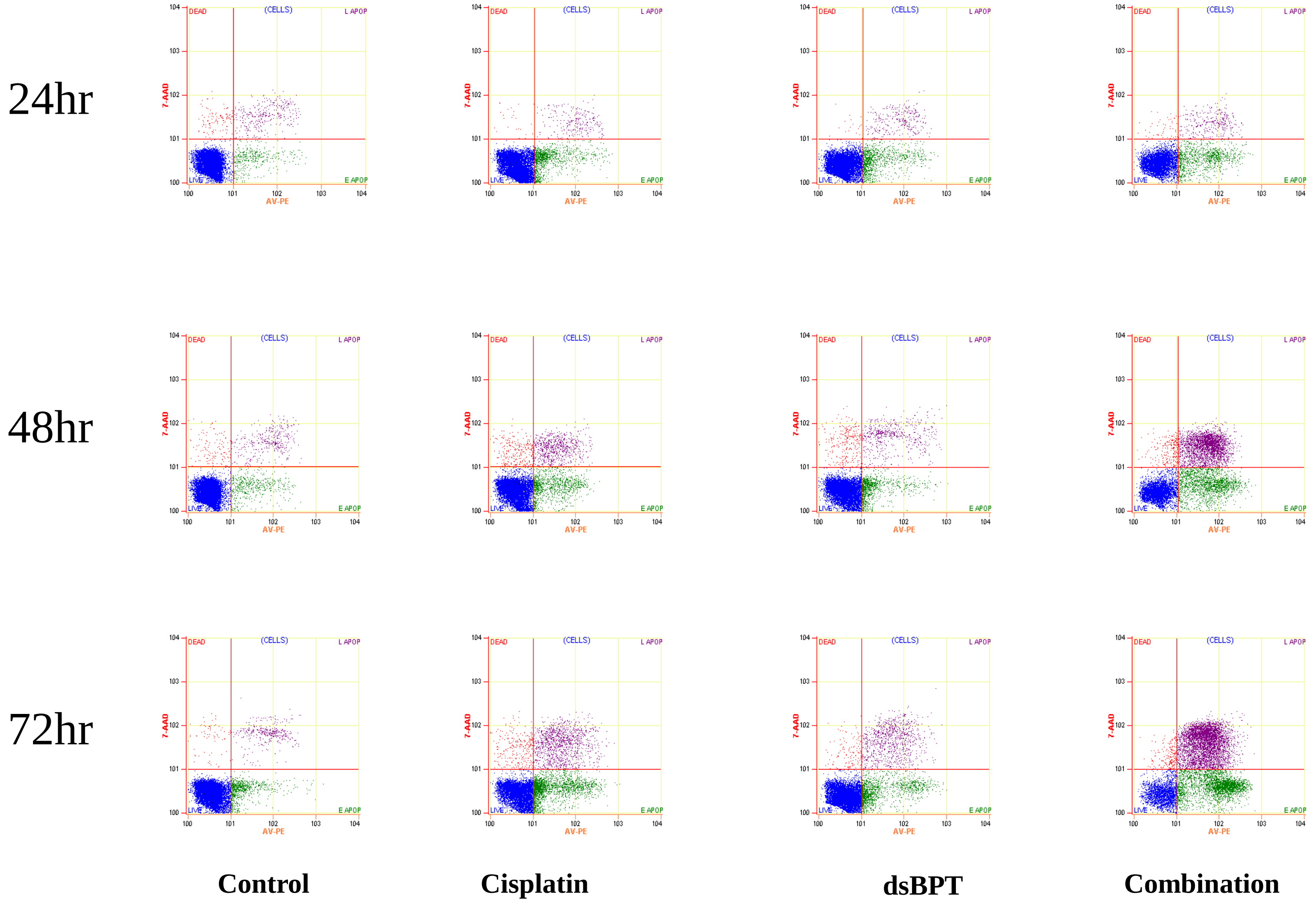

Supplementary Fig S1
