# Supplementary figures and images for: Antitumor Activity of 2,9-Di-Sec-Butyl-1,10-Phenanthroline
Source: PLoS One. 2016 Dec 29;11(12):e0168450. doi: 10.1371/journal.pone.0168450 (PMC5199049; doi:10.1371/journal.pone.0168450)

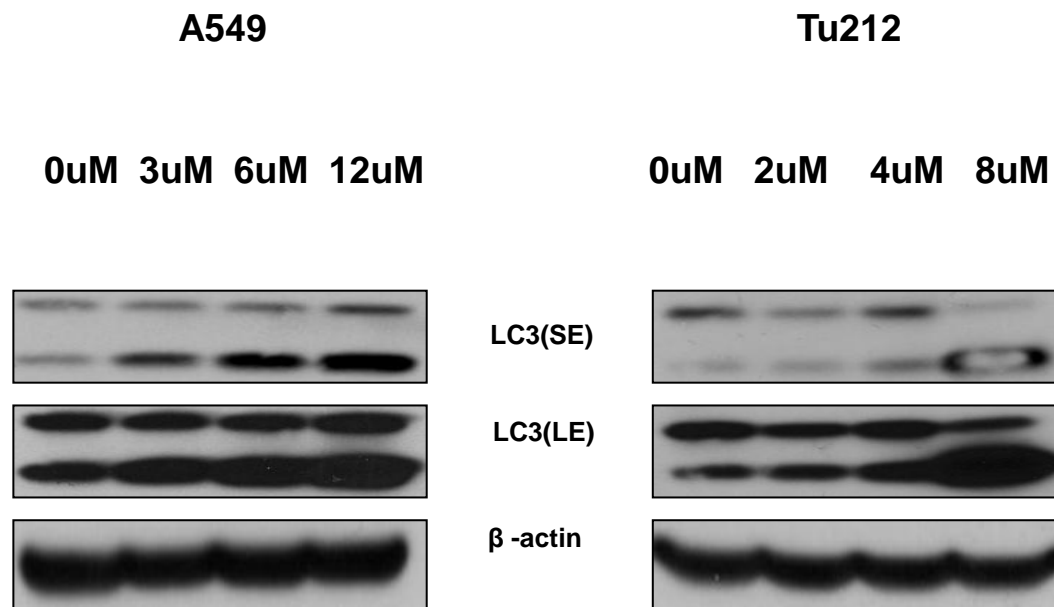

Supplement: S2 Fig — dsBPT treatment induced LC3 expression in both A549 and Tu212 cell lines. (SE stands for short exposure, LE stands for long exposure). (PDF) [file pone.0168450.s002.pdf]

A549

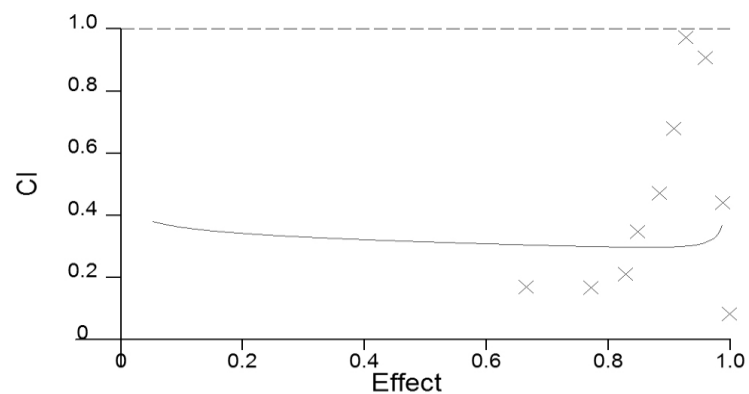

| Drug             | CI Values at |         |         |
|------------------|--------------|---------|---------|
|                  | ED50         | ED75    | ED90    |
| Cisplatin        | N/A          | N/A     | N/A     |
| dsBPT            | N/A          | N/A     | N/A     |
| Mixture<br>(1:1) | 0.31536      | 0.30140 | 0.29873 |

Supplement: S3 Fig — dsBPT with cisplatin synergistically enhanced the inhibitory sensibility of the both drugs as indicated by CIs which are less than 1.0. Figure is generated using CalcuSyn 2.0 software (Biosoft). (PDF) [file pone.0168450.s003.pdf]

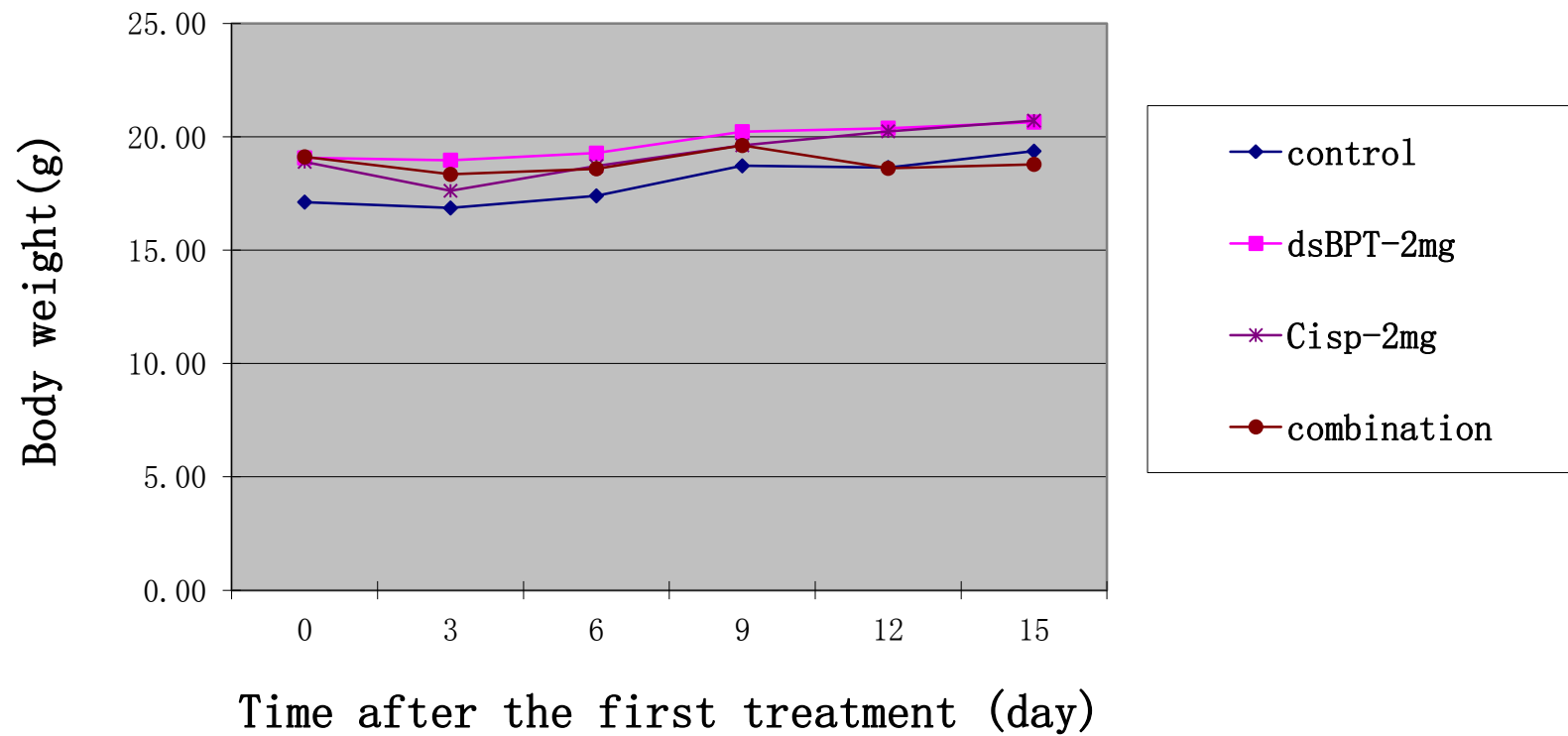

**Supplementary Figure S4**

Supplement: S4 Fig — No bodyweight loss was observed when mice were treated with 2mg/Kg dsBPT, cisplatin, and combination of the two agents with the same concentration. (PDF) [file pone.0168450.s004.pdf]
